# Digital Health Literacy during COVID-19: gender differences from a Florentine University experience

**DOI:** 10.1093/eurpub/ckac131.348

**Published:** 2022-10-25

**Authors:** A Guida, C Morittu, V Gallinoro, V Ferro Allodola, O Okan, K Dadaczynski, C Lorini, V Lastrucci, G Bonaccorsi

**Affiliations:** 1 School of Specialization in Public Health, University of Florence, Florence, Italy; 2 Department of Health Science, University of Florence, Florence, Italy; 3 Health Literacy Laboratory, University of Florence, Florence, Italy; 4 Department of Sport and Health Sciences, Technical University Munich, Munich, Germany; 5 Department of Nursing and Health Science, Fulda University of Applied Sciences, Fulda, Germany; 6 Epidemiology Unit, Meyer Children’s University Hospital, Florence, Italy

## Abstract

Gender appears to be a strong predictor of online health information-seeking behaviour (OHISB). Gender differences in OHISB have been studied in different countries with different results, but no studies investigated gender-specific behaviour among University students during the COVID-19 pandemic, which has brought with it a consequential infodemic. We sought to investigate any gender differences in OHISB in the period between the first and the second wave of the COVID-19 pandemic. A questionnaire promoted by the COVID-HL network was administered to 2996 students of all the courses at the University of Florence. It included existing validated scales adapted to the COVID-19 pandemic and newly developed scales. Gender differences were tested using the χ2 test or the Mann-Whitney U test, where appropriate. Male students reported a higher score in DHL than females (p < 0.001). However, female students reported using more often different sources for online information seeking (p < 0.05, except for YouTube), searching more corona-related topics (p < 0.05, except for economic and social consequences) and considering ‘‘very important'’ each item in the ‘‘Importance of internet information search'’ section (p < 0.05). Furthermore, female students are more likely to be ‘‘often dissatisfied'’ or ‘‘partly satisfied'’ with information about COVID-19 (p < 0.001) and to search more often for information for themselves and other people. Our study confirmed that gender could affect the way students search for health information on the Internet. Since students, in particular females, have been affected by stress and anxiety during the pandemic, these findings could help institutions to promote gender-specific education programmes to improve students’ DHL and their mental health outcomes, as well as to provide health information that fit specific gender needs.

**Key messages:**

• Gender influences how university students search on the Internet for health information.

• This should guide institutions to better address educational programmes to improve their Digital Health Literacy.

